# The Impact of Socioeconomic Factors and Geriatric Syndromes on Frailty among Elderly People Receiving Home-Based Healthcare: A Cross-Sectional Study

**DOI:** 10.3390/healthcare10102079

**Published:** 2022-10-19

**Authors:** Antonia Aravantinou-Karlatou, Savvato Kavasileiadou, Simeon Panagiotakis, Chariklia Tziraki, Wafa Almegewly, Emmanouil Androulakis, Christos Kleisiaris

**Affiliations:** 1 School of Social Sciences, Hellenic Open University, 26335 Patras, Greece; 2 Department of Community Health Nursing, College of Nursing, Princess Nourah bint Abdulrahman University, Riyadh 11671, Saudi Arabia; 3 Geriatric Clinic, University General Hospital of Heraklion, 71500 Heraklion, Greece; 4 Institute of Agri-Food and Life Sciences, University Research Centre, Hellenic Mediterranean University, 71410 Heraklion, Greece; 5 Mathematical Modeling and Applications Laboratory, Hellenic Naval Academy, 18538 Pireas, Greece; 6 Department of Nursing, School of Health Sciences, Hellenic Mediterranean University, 71410 Heraklion, Greece

**Keywords:** frailty, dementia, comorbidity, depression, homecare, aging, elderly people

## Abstract

Purpose: To evaluate frailty and its relationship with geriatric syndromes in the context of socioeconomic variables. Patients and Methods: In this cross-sectional study, elderly people aged 65 years old and over who received homecare in the reference region of Crete, Greece, were enrolled. Geriatric syndromes such as frailty, dementia, and depression were evaluated using the SHARE-Frailty Index (SHARE-Fi), the Montreal Cognitive Assessment (MoCA), and the Geriatric Depression Scale (GDS), respectively. Level of education, annual individual income, disability in Activities of Daily Living (ADL) and homebound status were also assessed as ‘socioeconomic factors.’ Results: The mean age of 301 participants was 78.45 (±7.87) years old. A proportion of 38.5% was identified as frail. A multiple logistic regression model revealed that elderly people with cognitive dysfunction were more likely to be frail (OR = 1.65; 95% CI: 0.55–4.98, *p* = 0.469) compared to those with normal cognition, but this association was not significant. Although elderly people with mild depression were significantly more likely to be frail (OR = 2.62; CI: 1.33–5.17, *p* = 0.005) compared to those with normal depression, the association for elderly people with severe depression (OR = 2.05, CI: 0.80–5.24, *p* = 0.134) was not significant. Additionally, comorbidity (OR = 1.06, CI: 0.49–2.27, *p* = 0.876) was not associated with frailty, suggesting that comorbidity is not a risk factor for frailty. In addition, patients with mild depression were significantly more likely to report frailty (OR = 2.62, CI:1.33–5.17, *p* = 0.005) compared to those with normal depression, whereas elders with an annual individual income (>EUR 4500) were less likely to be frail (OR = 0.45, CI: 0.25–0.83, *p* = 0.011) compared to those with <EUR 4500 per year. Conclusions: Our data analysis shows that higher annual individual income and mild depression were independently associated with frailty, suggesting that a lower poverty threshold and mild depression are risk factors for frailty.

## 1. Introduction

Frailty is a clinical geriatric syndrome characterized by increased vulnerability to adverse events such as delirium, falls or disabilities, when an elderly person is exposed to stressors, physical or psychosocial [1]. Although there is no consensus definition for frailty, nosologically it includes two domains: physical frailty (weight loss, exhaustion, low physical activity, reduced strength) and social frailty (social roles, social networks, and social activity). Interestingly, social frailty may precede and lead to the development of physical frailty [2]. While other aspects of frailty include socioeconomic and psychosocial factors such as a loss of resilience in cognition [3].

The prevalence of frailty varies among countries due to the diverse financial development of each country. European countries with lower socioeconomic status tend to have higher frailty incidence [4]. In Greece, the prevalence of frailty was found to be 14.7% and 44.9% for pre-frail elderly people aged 65 years or older, following the SHARE-Fi criteria [5]. Clinically, frail elderly people present a faster and more severe decline in cognitive function compared to pre-frail people. Frailty is associated with complex processes in the body, although it is not yet clarified if there is a difference between frail elderly people or if all these pathological changes are a result of the normal aging process and not necessarily of frailty [6]. Aging causes a gradual decline in physical activity, and this is associated with frailty syndrome as it accelerates and inevitably leads to malfunctions in several systems [7].

It has been suggested that frailty is associated not only with its phenotypic characteristics (biological, psychological, and social factors) but also with age-related syndromes such as dementia and depression. This association means that changes in the behavior of elderly people may decrease their functionality and thus lead to frailty. Simultaneously, elderly people with impaired physical functionality are more likely to develop depression [8]. Studies have found that severe depression is a risk factor for frailty and one of the main difficulties presented in older individuals’ in-home care. Furthermore, this interaction has not been fully clarified yet, but studies have shown that depression and frailty have common features and may also share a common pathology. Frailty is also associated with socioeconomic factors, such as low levels of education [9], reduced access to health services, age, gender, low economic status, or living below the poverty line [10].

Since 2001, “Help at Home” homecare programs have been established across Europe, including Greece, and supported by the Greek Ministry of Health and funded by the European Union, targeting elderly people who need help at home, particularly medical and nursing person-centered care, social support, solidarity network, and prevention of diseases, loneliness, social isolation, and institutionalization [11]. However, in Greece, only a few studies have been conducted in these programs focusing on frailty. Our previous published study in Greece in Help at Home programs showed that frailty and cognitive dysfunction, including depression were the most important risk factors for disability in ADL, leading to loss of independence for the beneficiaries of these programs. Specifically, depression was associated with cognitive dysfunction (a dependent variable) regardless of socioeconomic variables [12]. On the other hand, the impact of socioeconomic variables associated with frailty has not been fully investigated, placing frailty as a ‘dependent’ variable in data analysis. Within this framework, we sought to explore the effect of socioeconomic factors and geriatric syndromes on frailty.

### Aim

This study aimed to identify the incidence of frailty and geriatric syndromes such as cognitive function (decline), depression, comorbidity, and socioeconomic factors and their effect on frailty among the elderly population receiving home-based healthcare services.

## 2. Material and Methods

### 2.1. Study Design and Participants

This is a cross-sectional study of home-dwelling older people in Crete, Greece. The study was conducted as part of clinical interventions designed by the Institute of Agri-Food and Life Sciences, University Research Centre, Hellenic Mediterranean University in collaboration with the MSc Program “Management of Aging and Chronic Diseases” of the Hellenic Open University. In addition, this study is as part of preventive actions committed to the European A3 Action Group “Lifespan Health Promotion & Prevention of Age-Related Frailty and Disease” [13].

Following an invitation by the local municipal authorities, 546 elderly (65 years old and over) who are registered members of “Help at Home” programs were invited to participate in a door-to-door screening for frailty incidence and socioeconomic status program in the Reference Region of Crete, Greece. People who agreed to take part in the study were enrolled in the screening for frailty program and were tested for frailty and its risk factors, such as cognitive function, depression, annual income, etc. Data were collected from March to May 2019.

All registered members at ‘Help at Home’ programs who were aged 65 years old and above were recruited, but a large number of those were excluded due to their difficulty to provide the requested information using the screening tools. Additionally, due to communication impairments (severe dementia, and post-stroke and other severe comorbidities), completely bedridden, and those who refused to participate. Finally, only 301/546 older people participated and were incorporated in the statistical analysis (response rate of 55.1%).

### 2.2. Instruments

#### 2.2.1. Frailty Assessment

The *SHARE-Frailty Instrument (SHARE-Fi)*, a specific tool for the screening of physical frailty, was used [14]. The validity and reliability of the SHARE-Fi based on mortality predictors (women 0.79 and men 0.77) was confirmed by statistical comparisons of the AUCs methods and the complex Flx index based on Comprehensive Geriatric Assessment. Additionally, SHARE-Fi was created based on Fried’s phenotypic frailty, using the adapted phenotypic variables such as muscle weakness, exhaustion, unintentional weight loss, slowness, and low physical activity. Originally, the SHARE-Fi scoring system classified frailty status as follows: 0 points mean non-frail, 1–2 points mean pre-frail and 3–5 points mean frail. In this study, an algorithmic web-based calculator tool was used to provide a continuous frailty score and enable a classification into three phenotypic frailty categories: non-frail, pre-frail, and frail.

#### 2.2.2. Geriatrics Syndromes

The validated (sensitivity 0.9) screening tool, Montreal Cognitive Assessment scale (MoCA) was used to identify cognitive function [15]. The Greek validated version was used presenting sensitivity 0.82 and specificity 0.90, in a sample of 710 Greek patients [15]. The overall score of MoCA is ranging from 0 to 30, and patients with scores less than (<26) were considered patients with a high probability of having cognitive dysfunction or a decline in cognition. In completing this scale, the participants have to perform visual capacitive exercises such as clock drawing, cube copying, problem-solving, and answering in memory and orientation questions.

The Greek version of the Geriatric Depression Scale (GDS) was used to assess geriatric depression (sensitivity of 92% and specificity of 95%). The short form of GDS is a standardized scale and consists of 15 closed-ended queries (YES-NO), with a total score range of 0–15. A score between 6–10 indicates mild depression, whereas a higher score (11 or overscore) is indicative of severe depression [16].

The impact of comorbidity on frailty was assessed with the use of the Charlson Comorbidity Index (CCI) and was tested for possible confounding effects [17]. The CCI was used to assess the prognostic burden of comorbid diseases such as myocardial infarction, diabetes, etc. Data were entered into the web-based calculator and latent classes of comorbidity. In the present study, data were analyzed following the ordinal categories: 0–1 in the absence of comorbidity, 2–4 mild to moderate comorbidity, and ≥5 severe comorbidity [18].

#### 2.2.3. Socioeconomic Factors

Given that frailty is a risk factor for disability in Activities of Daily Living performance [19], affecting the social life of older people [20], and leading to a lifelong disadvantage [21], the Greek version a the Barthel Scale was used (reliability Cronbach-a 0.87 & 0.95 universal). Particularly, the Barthel scale assesses the level of functional independence in the domains of personal care and mobility. More specifically, it measures the abilities of a patient (what could do and what not) [22]. The overall scores of this scale ranges from 0–20, in the following categories (dependent, semi-dependent, and independent), with lower values indicating increased disability, reduced functionality, or loss of independence following Collin and his colleagues’ classifications [23].

The homebound status of older people was also assessed for potential confounding effects. Specifically, homebound status refers to the ability of a person to leave home for the last month or at most once a week in the last month. Semi-homebound means going out twice a week for the last month but with help or difficulty. Not being homebound means going out at least twice per week without help or difficulty [24].

In addition, an improvised questionnaire was used, so demographic and social characteristics of older people, such as gender, age, marital status, education, and annual income, were collected. It is worth mentioning that an annual individual income of fewer than EUR 4,500 for the economic year of 2019 was considered the “poverty threshold” of the participants according to the official socioeconomic data of the Hellenic Statistical Authority [25]. 

### 2.3. Ethical Consideration

This study was ethically approved by the Scientific Committee of the Hellenic Open University, Greece and the Municipality authorities “Help at Home” program of Crete (Pr No 297, 2019). The data collected were anonymous. Participants were kindly asked to participate after given their informed consent, and it was clearly underlined that their participation was voluntary, and they could withdraw at any time during the study.

### 2.4. Data Analysis

Data were coded and analyzed using the Statistical Package for Social Science IBM SPSS 24.0. Descriptive statistics were generated as appropriate for each variable. Initially, categorical variables were summarized as frequencies and percentages, while continuous variables were presented as mean, SD and median (IQR). Additionally, Shapiro–Wilk’s test was used to assess the normality of quantitative variables. The existence of normality was also confirmed or rejected by the visual overview of the respective histograms, the normal Q-Q charts, and the box-plots of the variables. In all cases considered necessary, exact tests or Monte Carlo simulations (10,000 samples), were used in the statistical tests. Additionally, logistic regression models were used to explore the impact of socioeconomic factors (annual individual income, level of education, disability in ADL and homebound status) and geriatric syndromes (depression, cognitive dysfunction, and comorbidities) on frailty and the effect of potential confounders. Data analysis was presented using odds ratios (OR) with 95% confidence intervals. A *p*-value < 0.05 was preset as statistically significant.

## 3. Results

### 3.1. Demographic Data of the Participants

Social-demographic data of the participants are presented in Table 1. The mean age of the 301 participants was 78.45 ± 7.87 years and (81.4%) were uneducated (primary school). The prevalence of geriatrics syndromes presented as follows; frailty (38.5%), cognitive dysfunction (87.8%), severe depression (13.6%), and severe comorbidity (70.6%), whereas 7.2% of the elderly people lost their disability in ADL and 18.8% were homebound.

### 3.2. Distribution of the Socio-Economic Factors and Geriatrics Syndromes according to Gender and Frailty Classification

Table 2 shows the distribution of frailty (3 categories) in participants with geriatric syndromes and socioeconomic factors according to gender. Briefly, frailty was more frequent in females (25.5%) with cognitive dysfunction and aged > 80 years old (13.6%), compared to males (10.8%) with cognitive dysfunction and aged > 80 years old (7.3%). Frailty was also more frequent in females with lower annual individual income (15%) and low formal education levels (23%), compared to males with lower annual individual income (7.1%) and low formal education levels (10.5%).

### 3.3. The impact of Geriatric Syndromes on Frailty

**Figure 1:**
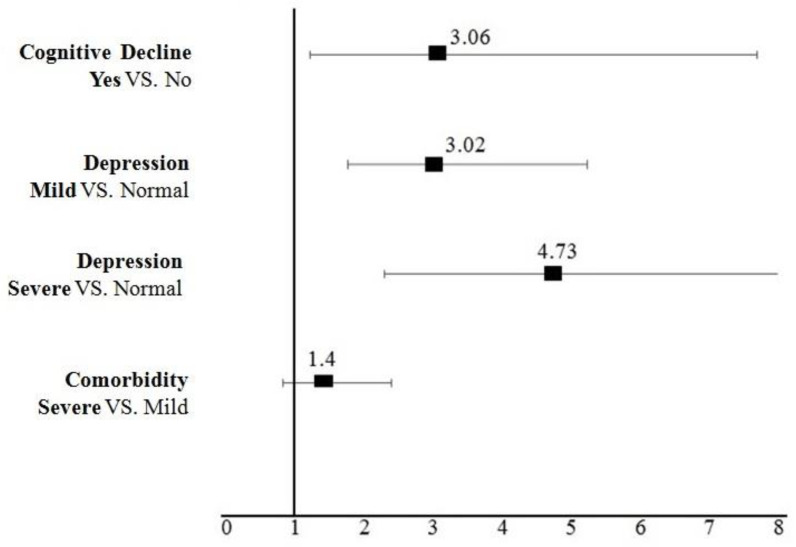
The impact of Geriatrics Syndromes on Frailty (univariate model analysis). Notes: An OR > 1 indicates a greater likelihood of elders having (Yes, Mild, Severe, VS., etc.) to be frail. Bars represent 95% Confidence Intervals (CI). Abbreviations: OR, odds ratio.

Univariate analysis showed that elderly people with cognitive dysfunction were significantly more likely to be frail compared to those with normal cognition (OR = 3.06; 95% CI: 1.21–7.69, *p* = 0.017). In addition, elderly people with mild depression were significantly more likely to be frail compared to those with normal depression (OR = 3.02; 95% CI: 1.75–5.22, *p* < 0.001). Similarly, patients with severe depression (OR = 4.73; CI: 2.28–9.80, *p* < 0.001) were more likely to be frail compared to those with normal depression. Participants with severe comorbidity were more likely to be frail (OR = 1.40; CI: 0.82–2.39, *p* = 0.213) compared to patients with mild comorbidity, but this association was not significant—(Figure 1 and Table S1).

**Figure 2:**
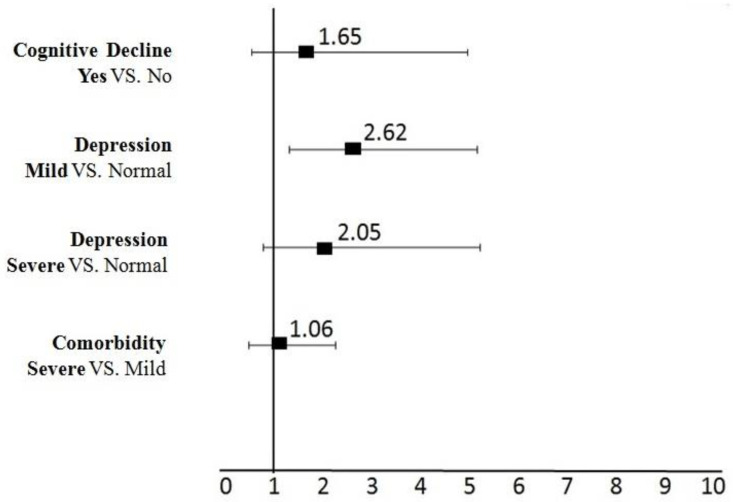
The impact of geriatrics Syndromes on Frailty (Multi-variate model analysis). Adjusting OR for gender, age, annual individual income, smoking, and educational level. Notes: An OR > 1 indicates a greater likelihood of elders having (Yes, Mild, Severe, VS., etc.) to be frail. Bars represent 95% (CI). Abbreviations: OR, odds ratio.

After adjusting for confounding effects (gender, age, annual individual income, smoking, and educational level), multiple logistic regression model revealed that elderly people with cognitive dysfunction were more likely to be frail (OR = 1.65; 95% CI: 0.55–4.98, *p* = 0.469) compared to those with normal cognition, but this association was not significant. Although elderly people with mild depression were significantly more likely to be frail (OR = 2.62; CI: 1.33–5.17, *p* = 0.005) compared to those with normal depression, the association for elders with severe depression (OR = 2.05, CI: 0.80–5.24, *p* = 0.134) was not significant. Additionally, comorbidity (OR = 1.06, CI: 0.49–2.27, *p* = 0.876) was not associated with frailty—(Figure 2 and Table S1).

**Figure 3:**
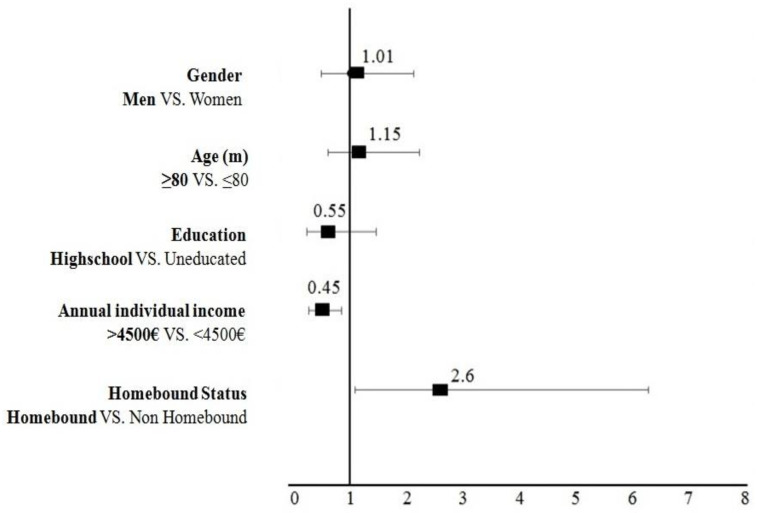
The impact of Socioeconomic factors on Frailty (Multi-variate model analysis). Notes: An OR > 1 indicates a greater likelihood of elders (Men, >80, Highschool, Homebound VS., etc.) to be frail. Bars represent 95% (CI). Adjusting OR for gender, age, annual individual income, smoking, and educational level. Abbreviations: OR, odds ratio.

In Figure 3, we present the associations between socioeconomic factors and frailty, applying multi-variate logistic regression model. In brief, frailty did not significantly differ between males and females (OR = 1.01; 95% CI: 0.48–2.11, *p* = 0.972), and those aged >80 (OR = 1.15; CI: 0.60–2.22, *p =* 0.658) and aged <80. Elderly people with higher formal education levels were less likely to be frail (OR = 0.55; CI: 0.21–1.45, *p* = 0.233) but this association was not significant. However, elderly people with higher (>EUR 4500) annual individual income (OR = 0.45; CI: 0.25–0.83, *p* = 0.011), were less likely to be frail compared to those with limited formal education (uneducated) and those with <EUR 4500, respectively. As expected, homebound elderly people were significantly more likely to be frail (OR = 2.60; CI: 1.07–6.28, *p* = 0.03) in comparison to non-homebound elders. (See Table S1).

## 4. Discussion

To our best knowledge, this is the first study in Greece that investigated the impact of socio-economic factors and geriatric syndromes on frailty in elderly people who received home-based healthcare. Our study began as a part of our commitment to the A3 Action Group of the European Innovation Partnership on Active and Healthy Aging [13]. The current study showed that higher annual individual income and higher formal education levels were associated with frailty. In addition, geriatric syndromes (dementia-related cognitive dysfunction and depression), particularly mild and severe depression, were significantly associated with frailty. The current study found that comorbidity was not associated with frailty, even after adjusting for potential confounding effects.

The main finding of the present study was that annual individual income was associated with frailty. This finding suggests that elderly people with higher individual incomes were less likely to present with frailty. In other words, having ‘money’ seems to act as a protective factor that delays the progression of frailty. In agreement with the results of the present study, similar findings were reported that the low economic status in elderly people was associated with the presence of frailty (OR = 1.07), with the use of the CSHA frailty scale [10].

However, each European country uses different financial indexes for the definition of the poverty threshold. Given the current financial crisis in Greece and its impact on the daily lives of the elderly, any comparison among countries should be avoided. Likewise, each study uses different screening tools, and thus only the possible explanations of these associations can be provided by describing the possible epidemiological differences among countries. In this line, data from several European countries (where the CHS frailty scale was applied) concluded that financial status had a different impact due to the various levels of economic development of each country [4]. In addition, recent primary research on frailty showed that poverty status in elderly people is associated with higher frailty levels, suggesting that poverty can be considered an important social factor in health as it corresponds to a complex of accumulated risk factors [26]. One of the main factors associated with frailty is depressive symptoms (OR: 0.91) which happen due to poverty factors [27].

We also found that other socioeconomic factors such as higher education levels were associated with frailty, suggesting that higher education levels may act as a protective factor, although the association was not significant. Additionally, it has previously been reported that a high level of education has a significant relationship with frailty (OR = 2.30) [28], thus decreasing the probability of having frailty and that the lack of educational qualifications (OR: 1.19) was a significant risk factor for frailty onset [7].

Another important finding of the present study was that geriatric syndromes, particularly mild depression, were significantly associated with increased odds of frailty in our participants. This association was not significant for elderly people with severe depression, even after adjusting for confounding effects. A possible explanation for this association could be that the recent financial crisis in Greece may have affected the daily lives of elderly people, and thus depression is a normal part of their lives. In other words, our sample may experience depressive symptomatology at a mild level in their daily life during this period (financial crisis) in Greece. Yet, there is no study available conducted in Greece to provide comparative data.

However, several other studies support the assumption that frailty shares common symptoms with depression [29,30] and a recent study conducted on elderly people showed that those who had depressive symptoms were at a higher risk for presenting with frailty [31]. Data from a recent meta-analysis showed that elderly people with depression had an increased odds of having frailty (OR = 4.07) presenting a significant interaction between depression and frailty in elderly people [32].

Additionally, elderly people with depression may affected by dementia, as these two geriatric syndromes often coexist [30]. In our study, elderly people with dementia-related cognitive dysfunction were presented with a higher probability of having frailty (OR = 3.06), but after applying logistic regression analysis, this relationship was not significant. Data from our previous work in Greece (192 home care participants ≥65) showed that dementia and frailty were strongly associated [12].

However, the relationship was attributed to the increased prevalence of depression in our sample. Other factors such as socio-economic may act as confounders in this association independently of dementia. In comparison, studies conducted worldwide showed that elderly people with cognitive dysfunction have a significant chances of presenting frailty (OR = 1.78) [33]. Similarly, other studies showed that cognitive decline was significantly associated with an increased odds of frailty (OR = 1.15) [9]. So, another explanation could be that higher levels of depressive symptomatology may come from elderly people suffering from both dementia and frailty.

With regard to the comorbidity index (CCI), it does not seem to have a significant relationship with frailty. In this study, comorbidity was not associated with frailty. In an argument with our findings, recent studies have shown an increased odds for frailty development when the comorbidity is present (OR = 1.90) [34] and (OR = 3.16) [33]. In other studies, elderly people’s data demonstrated the high incidence rate of comorbidity, especially when geriatric syndromes, particularly dementia and depression, coexist.

## 5. Future Implications

In Greece, both frailty and geriatric syndromes remain undiagnosed in elderly people, especially those receiving home-based healthcare, usually provided by their family members and less by primary healthcare professionals. Noteworthy, there are no integrated care standards and no protocols for homecare, apart from those provided by the local health care units for elderly people. In addition, most of the health professionals in these programs are not sufficiently trained and qualified in the assessment or evaluation of the early diagnosis of frailty and gerontology in general. Therefore, the need to improve the existing primary health system in Greece, including the education and training of health professionals in designing person-centered care, is apparent. Consequently, policymakers and vocational educational training providers have to focus on the development of geriatric nursing protocols encompassing skills and competencies related to the early recognition of frailty and dementia-related symptoms, thus preventing frailty and geriatric syndromes [35,36].

## 6. Limitations

Despite the several strengths, this study had certain limitations, the main one being the low response rate (55.1%). Although the original aim of the study was to screen all registered members of the ‘Help at Home’ programs, a large number of elderly people were not enrolled due to communication impairments (severe dementia and post-stroke patients, completely bedridden, and elderly people who refused to participate). Nevertheless, this is a common bias in home-oriented original studies, prevalence rates were used only as variables in the statistical analysis. Another limitation was that, despite the SHARE-Fi, a mix-performance reliable tool for Greek elderly people, data collection came from self-reports (participants’ witnesses) and thus, may have a high degree of bias and lead to unsafe conclusions.

## 7. Conclusions

The results suggested that socioeconomic factors such as higher annual individual income were strongly and independently associated with frailty, suggesting that a lower poverty threshold is a risk factor for frailty in elderly people. As regards geriatric syndromes, mild depression was associated with frailty in our elderly sample. Subsequently, health professionals caring for elderly people, especially family and community nurses, may focus on the early recognition of frailty and depression symptomatology. Therefore, not only the use of validated screening tools in daily clinical practice but also a comprehensive geriatric assessment may be the best and/or ‘good practice’ for the prevention of frailty in the framework of active and healthy aging.

## Figures and Tables

**Table 1:** Descriptive statistics of the study participants (n = 301).

	Mean ± SD, Median (IQR)	
Age (years)	78.45 ± 7.87, 79.00 (11.00)	
	**N**	**%**
Gender		
Male	111	36.9
Female	190	63.1
Annual individual Income		
≤4500	133	45.4
>4500	160	54.6
Educational Level		
* Uneducated	245	81.4
Highschool	36	12.0
Bachelor/MSc/PhD	20	6.6
Cognitive function ^a^		
Dysfunction (MoCA < 26)	253	87.8
Normal (MoCA ≥ 26)	35	12.2
Frailty status		
Frail	110	38.5
Pre-frail	130	45.5
Non-frail	46	16
Depression		
Severe (GDS 11+)	41	13.6
Mild (GDS 6–10)	106	35.2
Normal (GDS 0–5)	154	51.2
Comorbidity ^b^		
Severe (CCI ≥ 5)	207	70.6
Mild (CCI 2–4)	86	29.4
Normal (CCI 0–1)	0	0
Disability in ADL ^c^		
Dependent (Barthel ≤ 10)	21	7.2
Semi-dependent (Barthel 11–14)	25	8.5
Independent (Barthel 15+)	247	84.3
Homebound status ^d^		
Homebound	54	18.1
Semi-homebound	48	16.1
Non-homebound	196	65.8

Notes: Prevalence was given as actual numbers of elderly people (N) and percentages (%). ^a^ Cognitive Function: MoCA < 26 is indicative of cognitive dysfunction; ^b^ Comorbidity refers to the mean values of the CCI index and not to the actual number of illnesses; ^c^ Disability in ADL refers to the level of functional independence in the domains of personal care and mobility on performing Activities of Daily Living (ADL). Barthel ≤ 10 indicates that the person is dependent or “disabled”); ^d^ Homebound Status refers to the ability of a person to leave of home during the last month due to its illnesses. Homebound (able to leave home at least once a week in the last month); Semi-homebound: (able to get home about 2 times a week with help), Non-homebound: (about 2 times a week but without help). * Uneducated refers to having or showing a poor level of formal education (primary school). Abbreviations: GDS, Geriatric Depression Scale; CCI, Charlson Comorbidity index; Barthel Scale-Activities of Daily Living.

**Table 2:** Frequency of the socioeconomic factors with respect to frailty status (n = 301).

	Total		Females n (%)			Males n (%)		
			Frailty Status			Frailty Status		
	n	%	Non-Frail	Pre-Frail	Frail	Non-Frail	Pre-Frail	Frail
Frailty	286	100	18(6.3)	85(29.7)	79(27.6)	28(9.8)	45(15.7)	31(10.8)
Cognitive Function ^a^								
Dysfunction (MoCA < 26)	242	88	13(4.7)	74(26.9)	70(25.5)	16(5.8)	41(14.9)	28(10.2)
Normal (MoCA ≥ 26)	33	12	4(1.5)	10(3.6)	4(1.5)	11(4)	2(0.7)	2(0.7)
Age								
≤65–79	153	53.5	13(4.6)	51(17.8)	40(14)	18(6.3)	21(7.3)	10(3.5)
≥80	133	46.5	5(1.8)	34(11.9)	39(13.6)	10(3.5)	24(8.4)	21(7.3)
Depression								
Severe (GDS 11+)	41	14.3	0(0)	12(4.2)	19(6.6)	1(0.3)	3(1.1)	6(2.1)
Mild (GDS 6–10)	96	33.6	5(1.7)	24(8.4)	34(11.9)	6(2.1)	13(4.6)	14(4.9)
Normal (GDS 0–5)	149	52.1	13(4.6)	49(17.1)	26(9.1)	21(7.3)	29(10.1)	11(3.9)
Comorbidity ^b^								
Severe (CCI ≥ 5)	201	70.3	10(3.5)	59(20.7)	57(19.9)	18(6.3)	32(11.2)	25(8.7)
Mild (CCI 2–4)	85	29.7	8(2.8)	26(9.1)	22(7.7)	10(3.5)	13(4.5)	6(2.1)
Normal (CCI 0–1)	0	0.0	0(0)	0(0)	0(0)	0(0)	0(0)	0(0)
Disability in ADL ^c^								
Dependent (Barthel ≤ 10)	19	6.7	0(0)	5(1.8)	13(4.5)	0(0)	0(0)	1(0.4)
Semi-dependent (Barthel 11–14)	25	8.7	0(0)	6(2)	11(3.8)	0(0)	3(1.1)	5(1.8)
Independent (Barthel 15+)	242	84.6	18(6.3)	74(25.9)	55(19.2)	28(9.8)	42(14.7)	25(8.7)
Homebound Status ^d^								
Homebound	51	18	0(0)	14(4.9)	30(10.6)	0(0)	3(1.1)	4(1.4)
Semi-Homebound	42	14.9	0(0)	9(3.2)	19(6.7)	1(0.4)	3(1.1)	10(3.5)
Non-Homebound	190	67.1	17(6)	62(21.9)	30(10.6)	27(9.5)	37(13.1)	17(6)
Annual individual Income								
≤4500	125	44.6	8(2.9)	30(10.7)	42(15)	5(1.8)	20(7.1)	20(7.1)
>4500	155	55.4	9(3.2)	53(19)	36(12.9)	23(8.2)	25(8.9)	9(3.2)
Educational Level								
* Uneducated	230	80.4	14(4.9)	70(24.5)	66(23)	18(6.3)	32(11.2)	30(10.5)
Highschool	36	12.6	3(1.1)	10(3.5)	8(2.8)	7(2.4)	8(2.8)	0(0)
Bachelor/MSc/PhD	20	7	1(0.4)	5(1.7)	5(1.7)	3(1.1)	5(1.7)	1(0.4)

Notes: Data were given as actual numbers of elderly people (N) and percentages (%). ^a^ Cognitive dysfunction refers to MoCA < 26; ^b^ Comorbidity refers to the mean values of the CCI index and not to the actual number of illnesses; ^c^ Disability in ADL refers to the level of functional independence in the domains of personal care and mobility on performing Activities of Daily Living (ADL). Barthel ≤ 10 indicates that the person is dependent or “disabled”); ^d^ Homebound status refers to the ability of a person to leave or leaving the home during the last month due to its illnesses; Homebound (able to leave home at least once a week in the last month), Semi-homebound: (able to get home about 2 times a week with help), Non-homebound: (about 2 times a week but without help). * Uneducated refers to having or showing a poor level of formal education (primary school).

## Data Availability

The data presented in this study are available on request from the corresponding author. The data are not publicly available due to privacy restrictions.

## Supplementary Materials

The following supporting information can be downloaded at: https://www.mdpi.com/article/10.3390/healthcare10102079/s1, Table S1: The impact of socioeconomic factors and geriatric syndromes on frailty.
