# Supplementary material for: The Impact of Socioeconomic Factors and Geriatric Syndromes on Frailty among Elderly People Receiving Home-Based Healthcare: A Cross-Sectional Study
Source: Healthcare (Basel). 2022 Oct 19;10(10):2079. doi: 10.3390/healthcare10102079 (PMC9602759; doi:10.3390/healthcare10102079)
Supplement: Supplementary file 1 [file healthcare-10-02079-s001.zip › healthcare-1926499-supplementary.pdf]

healthcare MDPI

Supplementary

Table S1: The impact of socioeconomic factors and geriatric syndromes on frailty (n=301).

Logistic regression models									
	Univariable models			Multivariable model 1			Multivariable model 2		
	OR	95% CI	p-value	OR	95% CI	p-value	OR	95% CI	p-value
**Cognitive function**[a] (Moca)									
Dysfunction vs Normal (<26 vs ≥26)	3.06	1.21-7.69	0.017	1.59	0.58-4.34	0.357	1.65	0.55-4.98	0.469
**Depression** (GDS)									
Mild vs Normal	3.02	1.75-5.22	<0.001	2.16	1.15-4.04	0.016	2.62	1.33-5.17	0.005
Severe vs Normal	4.73	2.28-9.80	<0.001	2.33	0.99-5.47	0.052	2.05	0.80-5.24	0.134
**Comorbidity**[b] (CCI)									
Severe (CCI≥5) vs Mild	1.40	0.82-2.39	0.213	1.25	0.67-2.31	0.475	1.06	0.49-2.27	0.876
**Disability in ADL**[c]									
Barthel 11-15 vs Barthel 16-20	3.60	1.52-8.50	0.003	1.83	0.67-4.97	0.232	1.86	0.65-5.34	0.247
Barthel ≤10 vs Barthel 16-20	5.67	1.97-16.29	0.001	1.89	0.56-6.37	0.300	1.83	0.52-6.37	0.340
**Homebound status**[d]									
Semi-homebound vs Non-homebound	6.78	3.26-14.11	<0.001	4.55	2.05-10.06	<0.001	3.73	1.58-8.76	0.003
Homebound vs Non-homebound	6.08	3.11-11.88	<0.001	3.53	1.60-7.78	0.002	2.60	1.07-6.28	0.034
**CVD***									
Yes vs No	1.42	0.87-2.30	0.151	-	-	-	1.34	0.67-2.69	0.400
**Age** (years)									
≥80 vs <80	1.69	1.04-2.73	0.032	-	-	-	1.15	0.60-2.22	0.658
**Gender**									
Men vs Women	0.55	0.33-0.924	0.024	-	-	-	1.01	0.48-2.11	0.972
**Annual individual Income**									
>4500 vs <4500	0.41	0.25-0.68	<0.001	-	-	-	0.45	0.25-0.83	0.011
**Smoking**									
Smoker vs Non Smoker	0.41	0.16-1.03	0.060	-	-	-	0.69	0.20-2.41	0.568
Ex-Smoker vs Non Smoker	1.04	0.44-2.42	0.923	-	-	-	1.79	0.55-5.79	0.329
**Educational Level**									
Highschool vs Uneducated	0.39	0.17-0.91	0.030	-	-	-	0.55	0.21-1.45	0.233
Bachelor/MSc/PhD vs Uneducated	0.59	0.22-1.61	0.310	-	-	-	1.12	0.36-3.46	0.840

Notes: The impact of geriatric syndromes and socioeconomic factors on frailty taking into consideration possible confounding effects is examined in model 2 (Multivariate model), particularly multi-variate model 2 analysis after adjusting OR for CVD, age, gender, annual individual income, smoking, and educational level.

Abbreviations: OR, odds ratio. An OR>1 indicates a greater likelihood of elderly people having (Yes, Mild, Severe, VS. etc.) to be frail. Bars represent 95% Confidence Intervals (CI). [a]**Cognitive Function:** MoCA<26 is indicative of cognitive dysfunction; [b]Co**morbidity** refers to the mean values of the CCI index and not to the actual number of illnesses; [c]**Disability in ADL** refers to the level of functional independence in the domains of personal care and mobility on performing Activities of Daily Living (ADL). **Barthel ≤10** indicates that the person is dependent or "disabled"); [d]**Homebound status** refers to the ability of a person to leave of home during the last month due to its illnesses. Homebound (able to leave home at least once a week in the last month); Semi-homebound: (able to get home about 2 times a week with help), Non-homebound: (about 2 times a week but without help). **Abbreviations:** GDS, Geriatric Depression Scale; CCI, Charlson Comorbidity index; Barthel Scale -Activities of Daily Living. *CVD: Cardiovascular disease.
